# Predictors of small-for-gestational-age infants in gestational diabetes mellitus: the impact of metformin use

**DOI:** 10.1007/s00404-025-08029-z

**Published:** 2025-04-22

**Authors:** Sivan Farladansky-Gershnabel, Dina Lidsky-Sachs, Nur Abd El Qadir, Ronny Biton Ram, Tal Biron-Shental, Michal Kovo, Dorit Ravid

**Affiliations:** 1 Department of Obstetrics and Gynecology, Meir Medical Center, Kfar Saba, Israel; 2 Faculty of Medical and Health Sciences, Tel Aviv University, Tel Aviv, Israel

**Keywords:** Metformin, GDM, GDMA2, SGA

## Abstract

**Introduction:**

Gestational diabetes mellitus (GDM) affects 3%–25% of pregnancies worldwide, posing risks to maternal, fetal, and neonatal health. GDM is often associated with macrosomia and large-for-gestational-age (LGA) infants. However, the association between GDM and small-for-gestational-age (SGA) infants is less understood. This study aimed to identify predictors of SGA in women with GDM.

**Methods:**

This retrospective study included GDM patients (GDMA1 and A2) admitted to the fetal–maternal unit between 2014 and 2023. The study population was divided into those who delivered an appropriate for gestational age (AGA) neonate and those who delivered an SGA neonate (defined as birthweight < 10th percentile. Women with pregestational diabetes mellitus were excluded. Obstetric and neonatal outcomes were compared between the groups. A subgroup analysis focused on GDMA2 patients, comparing maternal and neonatal outcomes and treatment regimens (insulin and metformin use).

**Results:**

The study included 894 GDM patients. Compared to the AGA group (n = 712), the SGA group (n = 182) had lower maternal BMI (p = 0.02). Maternal age was comparable between groups. Rates of GDMA2 (30.2% vs. 23.4%, p = 0.07), and hypertensive disorders (7.1% vs. 5%, p = 0.21) did not differ significantly between the groups. The neonatal birthweight of the SGA infants was 2375 ± 432 g vs. 3021 ± 165 g in the AGA infants, (p = 0.005). The SGA group had a higher rate of CD due to NRFHR (27.4% vs. 18.4%, p < 0.01). Among GDMA2 patients (n = 222), more women in the SGA group (n = 55) were treated with metformin as compared to the AGA group (n = 167) (72.7% vs. 23.9%, p < 0.001). Multivariate regression analysis revealed that among GDMA2 patients metformin treatment was independently associated with SGA neonates OR 1.7, CI 1.18–1.35, p < 0.01).

**Conclusion:**

Metformin use in GDMA2 pregnancies may be linked to SGA neonates. The impact of metformin on fetal growth highlights the need for careful monitoring and individualized treatment strategies in managing GDMA2.

## What does this study add to the clinical work

**Table:** 

Metformin use in GDMA2 pregnancies was associated with a higher risk of delivering small for gestational age (SGA) infants compared to insulin therapy. These findings support a more individualized approach to pharmacologic management of GDM, particularly in patients with risk factors for fetal growth restriction.	

## Introduction

Gestational diabetes mellitus (GDM) represents a significant health challenge, with prevalence rates ranging from 3 to 25%, depending on ethnic composition, diagnostic criteria employed, and underlying population characteristics [1, 2]. This metabolic disorder, characterized by glucose intolerance first recognized during pregnancy, carries substantial risks for both maternal and fetal outcomes. Maternal complications include an elevated risk of hypertensive disorders, an increased likelihood of cesarean delivery (CD), and heightened susceptibility to subsequent type 2 diabetes mellitus (T2DM) [3, 4].

The primary fetal complication traditionally associated with GDM has been large-for-gestational-age (LGA) status, a consequence of fetal hyperinsulinemia triggered by maternal hyperglycemia. This condition predisposes infants to various complications, including macrosomia, shoulder dystocia, neonatal hypoglycemia, and long-term metabolic disorders [5]. However, emerging evidence suggests that GDM pregnancies may also result in small-for-gestational-age (SGA) infants, defined as birthweight below the 10 th percentile for gestational age [6, 7]. SGA infants face their own set of challenges, including respiratory distress, hypoglycemia, neurodevelopmental impairments, and increased risk of metabolic syndrome later in life [8].

When dietary interventions prove insufficient for glycemic control (GDMA1), pharmacological management becomes necessary (GDMA2) [9]. Insulin therapy, traditionally considered the gold standard due to its safety profile and its inability to cross the placental barrier, is recommended as the first-line treatment by several professional organizations, including the American Diabetes Association (ADA) and the American College of Obstetricians and Gynecologists (ACOG) [10, 12]. Alternatively, metformin has gained recognition as a viable treatment option, endorsed by the Society of Maternal–Fetal Medicine (SMFM) as a first-line treatment and by the National Institute for Clinical Excellence (NICE) as an adjunct or alternative to insulin [13, 14]. While metformin offers advantages in terms of administration, cost, and reduction in maternal weight gain, neonatal birth weight, and macrosomia [15, 16], its ability to cross the placental barrier raises questions about potential fetal effects through direct or indirect pathways, independent of glycemic control [17].

The optimal degree of glycemic control in GDMA2 patients remains a subject of debate, particularly concerning SGA risk. While strict glycemic control effectively prevents macrosomia, excessively stringent glucose targets may compromise fetal nutrient availability and growth, potentially contributing to SGA development [18, 19]. This study sought to identify predictive factors associated with SGA births among pregnant women with GDM, addressing an important gap in current maternal–fetal medicine research.

## Methods

### Study design and population

This retrospective cohort study was conducted using electronic health records from January 2014 to December 2023 of patients who were admitted during their pregnancy to the fetal–maternal unit at Meir Medical Center (MMC) and delivered at MMC. The main reasons for hospitalization included, among others, induction of labor, diabetes management, blood pressure stabilization, fetal monitoring, and observation. Included patients had GDM (A1 and A2) according to American Diabetes Association (ADA) guidelines [10], with a singleton pregnancy. Exclusion criteria included pre-existing diabetes, multiple gestations, and estimated fetal weight above the 90 th percentile. Patients with GDMA2 were managed with either insulin or metformin according to the American College of Obstetricians and Gynecologists (ACOG) guidelines [12] and standardized departmental protocols. The choice of insulin or metformin was made by the patient in conjunction with their physician’s recommendation. All included patients monitored glucose measurements using fingerstick blood glucose four times a day, including fasting measurements and one to two hours postprandial.

Patients were classified into two main groups based on neonatal birthweight: those who delivered small-for-gestational-age (SGA) infants (birthweight < 10 th percentile) and those who delivered appropriate-for-gestational-age (AGA) infants (birthweight between the 10 th and 90 th percentiles), according to Hadlock 4 sonographic growth curves [13].

### Data

The collected data included maternal and obstetrical characteristics. Maternal data included age, BMI (kg/m^2^), gestational age at delivery, pharmacological treatment, glucose control, and hypertensive disorders such as chronic and gestational hypertension and preeclampsia. Glycemic control was assessed based on glucose levels measured by the patient using a glucometer via fingerstick testing, both during fasting and postprandially. A maternal–fetal medicine specialist conducted the evaluation of glycemic control. All patients were referred for education and follow-up with a dietitian specializing in gestational diabetes. Obstetric characteristics included meconium-stained amniotic fluid, mode of delivery (vaginal delivery [VD], vacuum extraction [VE], or cesarean delivery [CD]), and whether CD was performed due to non-reassuring fetal heart rate (NRFHR) monitoring.

Early neonatal outcomes included Apgar score, phototherapy treatment, events of neonatal hypoglycemia, and length of neonatal hospitalization. Neonatal intensive care unit (NICU) hospitalization was recorded as well. A composite measure of adverse neonatal outcomes was defined as the presence of at least one of the following: NICU admission, neonatal hypoglycemia, need for phototherapy, or requirement for neonatal respiratory support.

Comparison of maternal characteristics, obstetric outcomes, and neonatal outcomes was performed between the SGA and the AGA groups. A subgroup analysis was performed for patients with GDMA2.

### Statistical analysis

Descriptive statistics were used to summarize maternal demographic characteristics, obstetric history, and neonatal outcomes. Between-group comparisons were conducted using Student’s t-tests for continuous variables and Chi-square or Fisher’s exact tests for categorical variables, with statistical significance set at p < 0.05. Logistic regression models assessed predictors of SGA in GDMA2 patients, adjusting for maternal age, gestational age, parity, BMI, hypertensive disorders, and metformin use, using SPSS (v.26).

## Results

During the study period, 894 women with GDM were included. Of them, 712 delivered AGA neonates and 182 delivered SGA neonates. Maternal BMI was significantly lower in the SGA group compared to the AGA group (27.31 ± 4.21 kg/m^2^ vs. 28.32 ± 5.2 kg/m^2^, p = 0.02). Mean maternal age, hypertensive disorders, and glycemic control levels were similar across groups. Gestational age at delivery was slightly lower in the SGA group (37.4 ± 1.1 weeks vs. 38.77 ± 1.67 weeks, p = 0.04) (Table 1).

**Table 1:** Maternal characteristics of the studied groups

	SGA group N = 182	AGA group N = 712	p-value
Maternal age (years)	32.1 ± 6.3	32.9 ± 4.8	0.35
BMI (kg/m^2^)	27.31 ± 4.21	28.32 ± 5.2	0.021
Gravidity	2.2 ± 1.4	1.9 ± 1.7	0.54
Parity	0.8 ± 1.0	1.2 ± 0.9	0.37
Smoking, n (%)	41 (12.5%)	8 (10.0%)	0.54
GA at delivery (weeks)	37.4 ± 1.1	38.77 ± 1.67	0.04
GDMA2 n (%)	55 (30.2%)	167 (23.4%)	0.073
Good glucose control	164 (90.1%)	629 (88.3%)	0.27
Hypertensive disorders*	13 (7.1%)	36 (5%)	0.21
Mean SBP	119.15 ± 18	121.4 ± 19.23	0.12
Mean DBP	76 ± 11.43	78.3 ± 14.1	0.63
*Mode of delivery*			
CD n (%)	68 (37.4%)	220 (30.9%)	0.39
CD NRFHR n (%)	50 (27.4%)	131 (18.4%)	< 0.01
VE n(%)	24 (13.1%)	58 (8.15%)	0.24
NVD n (%)	90 (49.4%)	434 (60.9%)	0.08

Data are presented as n (%) or mean ± SD

*BMI* Body Mass Index (kg/m^2^). *CD* cesarean delivery. Hypertensive disorders include gestational and chronic hypertension, as well as preeclampsia. *SGA* Small for Gestational Age; *AGA* Appropriate for Gestational Age; *GDMA2* Gestational Diabetes Mellitus A2; *NRFHR* Non-Reassuring Fetal Heart Rate; *VE* Vacuum Extraction; *NVD* Normal Vaginal Delivery. *SBP* and *DBP* systolic and diastolic blood pressure respectively

Neonatal birthweight in the SGA group was 2469 ± 365 g compared to 2971 ± 269 g in the AGA group (p = 0.005). Cesarean delivery rates due to non-reassuring fetal heart rate (NRFHR) were higher in the SGA group (27.4% vs. 8.15%, p < 0.01) (Table 1).

### GDMA2 group analysis

The GDMA2 subgroup included 222 women, of whom 55 (30.2%) delivered SGA neonates (Tables 2, 3). The SGA rate was higher among women treated with metformin compared to those treated with insulin (72.7% vs. 23.9%, p < 0.001). No significant differences were observed in the use of long-acting (Levemir) and short-acting (Novorapid) insulin between the SGA and AGA groups.

**Table 2:** Maternal characteristics and Obstetrics outcomes of GDMA2 patients

	SGA group n = 55	AGA group n = 167	p-value
Maternal age (years,SD)	32.6 ± 5.26	32.3 ± 5.3	0.29
G (mean ± SD)	2.2 ± 1.4	2.2 ± 1.6	0.38
P (mean ± SD)	0.8 ± 1.0	0.6 ± 0.9	0.17
Smoking	43 (12.5%)	8 (9.3%)	0.42
BMI (kg/m^2^, SD)	27.3 ± 3.4	28.2 ± 2.2	0.02
Gestational age (weeks, SD)	37.4 ± 2	38.49 ± 1.67	0.04
Metformin	40 (72.7%)	40 (23.9%)	< 0.001
Insulin Levemir	19 (34.5%)	48 (28.7%)	0.08
Insulin Novorapid	28 (5%)	11 (6.6%)	0.7
Good glucose control	49 (89%)	144 (86.2%)	0.3
Hypertensive disorders	5 (9%)	11 (6.5%)	0.34
*Mode of delivery*			
CD	19 (34.5%)	49 (29.3%)	0.49
CD urgent	40 (72.7%)	93 (55.6%)	< 0.01
CD NRFHR	27 (49%)	58 (34.7%)	< 0.01
VE	6 (10.9%)	16 (9.5%)	0.34
NVD	30 (54.5%)	101 (60.4%)	0.08

Data are presented as % or mean ± SD. *BMI* Body Mass Index (kg/m^2^). *CD* Cesarean Delivery. Hypertensive disorders include gestational and chronic hypertension, as well as preeclampsia. *SGA* Small for Gestational Age, while *AGA* Gestational Age. *GDMA2* Gestational Diabetes Mellitus requiring medication. *NRFHR* Non-Reassuring Fetal Heart Rate, *VE* Vacuum Extraction, and *NVD* Vaginal Delivery.

Compared to the AGA (GDMA2) group, neonatal birthweight in the SGA (GDMA2) group was significantly lower (2971 ± 269 g vs. 2469 ± 365 g, p = 0.005). The SGA (GDMA2) group was characterized by an increased rate of meconium-stained amniotic fluid (32.7% vs. 13.1%, p = 0.02), higher rates of cesarean delivery due to NRFHR (27.4% vs. 18.4%, p < 0.01), and neonatal phototherapy (72.7% vs. 59.8%, p = 0.03).

### Multivariate analysis

A multivariable analysis was conducted using logistic regression, including variables such as maternal age, BMI, gestational age, hypertensive disorders, and metformin use. In the GDMA2 cohort, metformin treatment emerged as an independent predictor of SGA, with an adjusted odds ratio of 1.7 (95% CI 1.18–1.35, p < 0.01). Lower maternal BMI also remained significantly associated with SGA risk (Table 3).

**Table 3:** Neonatal characteristics of GDMA2 patients

	SGA group - n = 55	AGA group- n = 167	p-value
Birthweight (mean ± SD)	2469 ± 365	2971 ± 269	0.005
5 min Apgar score ≤ 7	3 (5.4%)	4 (2.39%)	0.89
Phototherapy treatment	40 (72.7%)	100 (59.8%)	0.03
Neonatal hypoglycemia	4 (7.2%)	13 (7.78%)	0.896
Respiratory support, N (%)	6 (10.9%)	12 (7.2%)	0.57
Child NICU, N (%)	10 (18.1%)	12 (7.1%)	0.03
Neonate composite, N (%)	60 (27.2%)	137 (20.5%)	0.08
Gender- Male	23 (41.8%)	78 (46.7%)	0.3
Meconial amniotic fluid	18 (32.7%)	22 (13.1%)	0.02
Days of hospitalization (mean ± SD)	3.23 ± 1.97	2.91 ± 0.89	0.7

Data are presented as % or mean ± SD. GDMA2 indicates Gestational Diabetes Mellitus requiring medication. *NICU* neonatal intensive care unit

## Discussion

This study presents an association between metformin use in GDMA2 pregnancies (see Table 4) and an increased risk for SGA infants. Patients treated with metformin had a significantly higher risk of giving birth to an SGA infant compared to those treated with insulin (adjusted OR: 1.7, 95% CI 1.18–1.35, p < 0.01). Most of the patients with GDMA2 (72.7%) who gave birth to an SGA infant were treated with metformin, as compared to only 28.75% of those who gave birth to an AGA infant (p = 0.001).

**Table 4:** Risk factors for SGA among GDMA2 patients

Variable	Adjusted OR (95% CI)	p-value
Maternal age	1.2 (0.7, 1.1)	0.62
Gestational age	1.1 (0.9, 1.4)	0.44
Parity	0.6 (0.3, 2.1)	0.37
Maternal BMI	1.2 (0.6, 0.8)	0.02
Hypertensive disorders	1.9 (0.8, 5.5)	0.18
Metformin	1.7 (1.18,1.35)	< 0.01

The clinical significance of these findings is underscored by the elevated rate of urgent cesarean deliveries due to non-reassuring fetal heart rate (NRFHR) observed in the SGA group, coupled with an increased incidence of neonatal hyperbilirubinemia. These complications highlight the potential downstream consequences of SGA outcomes in GDMA2 pregnancies. Our results align with a recent observation in pregnant patients with type 2 diabetes (T2DM). A secondary analysis of the MiTy trial [11] by Feig et al. [21] revealed a high percentage of SGA births among women treated with metformin as compared to insulin only (12.9% vs. 6.6%). Comorbidities like chronic hypertension and/or nephropathy were found to augment the risk of SGA in T2DM patients treated with metformin. The present study did not find a correlation between hypertensive disorder and SGA.

Additionally, our findings are consistent with a comprehensive systematic review and meta-analysis comprising 33 studies with 4944 GDM patients randomized to receive insulin, metformin, or glyburide. The analysis demonstrated significantly reduced anthropometric measurements and diminished lean mass in neonates exposed to metformin in utero compared to those whose mothers received conventional insulin therapy [18]. While SGA rates were not specifically addressed in this analysis, the data suggested lower birth weights and decreased ponderal indices in the metformin group, corroborating previous reports of similar trends [16, 22, 23].

Furthermore, a systematic review and meta-analysis of seven trials comparing short- and long-term outcomes of metformin versus insulin treatment in GDM reported a relative risk of 1.2 for SGA infants, although this did not reach statistical significance (95% CI, 0.85 to 1.04) [24].

The biological plausibility of these findings may be explained by metformin's ability to cross the placenta and its potential effects on fetal and placental metabolism [18, 25]. The placenta and fetus express metformin transporters, exhibit high rates of aerobic metabolism, and are dependent on mature mitochondrial activity in the second and third trimesters, which are critical for fetal growth and nutrient transport. Metformin has the potential to inhibit mitochondrial activity, which can result in relative nutrient restriction that could adversely affect the growth or differentiation of fetal or placental tissue [26].

While metformin remains an accepted option for glycemic control in GDM [12, 13], our results support the recent American Diabetes Association (ADA) precautionary stance against metformin use in pregnancies complicated by hypertension, preeclampsia, or those at risk for fetal growth restriction [10].

This recommendation stems from theoretical concerns regarding potential fetal growth impairment and acidosis in the context of placental insufficiency. However, it is important to note that empirical evidence from human pregnancy outcomes has hitherto not demonstrated these theoretical adverse effects [27]. The present study, though retrospective and modest in the number of patients, may thus corroborate the ADA recommendation.

Our findings suggest that clinicians should consider a more nuanced approach to pharmacological intervention in GDMA2 pregnancies, particularly in cases where fetal growth patterns indicate potential concerns. Unlike cases of accelerated fetal growth where treatment intensification is warranted [20], management of SGA fetuses may require reconsideration of metformin as the primary therapeutic agent. Our observation needs further validation by prospective studies.

## Strengths and limitations

This study benefits from a robust sample size and the utilization of electronic health records, which facilitated comprehensive data collection and detailed analysis of maternal and neonatal outcomes. The single-institution design, with a consistent team of experienced clinicians, enhances the internal validity and reproducibility of the findings. Furthermore, the focused investigation of metformin’s impact on fetal growth contributes to the evolving literature, providing critical insights for optimizing the management of gestational diabetes mellitus (GDM).

However, inherent limitations must be acknowledged. The retrospective study design introduces potential biases related to data completeness and documentation accuracy. Additionally, reliance on electronic health records, while valuable for large-scale data analysis, may restrict the granularity of information on glycemic control, insulin titration, and other confounders that could influence fetal growth trajectories. The generalizability of findings may also be limited by the single-center setting, warranting further validation in diverse populations and prospective cohorts.

## Conclusions

In the management of pregnancies complicated by GDM requiring pharmacologic treatment (GDMA2), clinicians should carefully weigh the risk of small for gestational age (SGA) outcomes when prescribing metformin, particularly in women with low body mass index (BMI) or preexisting fetal growth restriction. An individualized approach, incorporating tailored glycemic targets, may mitigate the risks of both large for gestational age (LGA) and SGA, aligning with emerging evidence advocating for a nuanced, patient-specific strategy in GDM care. 

## Data Availability

No datasets were generated or analysed during the current study.

## References

[1] Rowan JA, Hague WM, Gao W, Battin MR, Moore MP (2008) Metformin versus insulin for the treatment of gestational diabetes. N Engl J Med 358(19):2003–2015 18463376 10.1056/NEJMoa0707193

[2] Niromanesh S, Alavi A, Sharbaf FR, Torabi R, Moosavi SG, Emdadi R, Habibollahi A (2012) Metformin compared with insulin in the management of gestational diabetes mellitus: a randomized clinical trial. Diabetes Res Clin Pract 98(3):333–339 10.1016/j.diabres.2012.09.031 23068960

[3] Spaight C, Gross J, Horsch A, Puder JJ (2016) Gestational diabetes mellitus. Endocr Dev 31:163–178 26824237 10.1159/000439413

[4] Kc K, Shakya S, Zhang H (2015) Gestational diabetes mellitus and macrosomia: a literature review. Ann Nutr Metab 66(Suppl 2):14–20 26045324 10.1159/000371628

[5] Langer O, Yogev Y, Most O, Xenakis EM (2005) Gestational diabetes: the consequences of not treating. Am J Obstet Gynecol 192(4):989–997 15846171 10.1016/j.ajog.2004.11.039

[6] Hilden K, Magnuson A, Hanson U, Simmons D, Fadi H (2020) Trends in pregnancy outcomes for women with gestational diabetes mellitus in Sweden 1998–2012: a nationwide cohort study. Diabet Med 37(12):2050–2057 32027045 10.1111/dme.14266

[7] Pan Y, Huang X, Jiang X (2024) Risk factors and prediction model for low birth weight infants born to women with gestational diabetes mellitus. Front Public Health 12:1432033 39469216 10.3389/fpubh.2024.1432033 PMC11514069

[8] Glueck CJ, Goldenberg N, Pranikoff J, Sieve L, Wang P (2004) Height, weight, and motor-social development during the first 18 months of life in infants of metformin-treated women with polycystic ovary syndrome. Hum Reprod 19(6):1323–1330 15117896 10.1093/humrep/deh263

[9] Kautzky-Willer A, Prager R, Waldhäusl W, Pacini G, Thomaseth K, Wagner O (1997) Pronounced insulin resistance and inadequate β-cell secretion characterize lean gestational diabetes during and after pregnancy. Diabetes Care 20(11):1717–1723 9353615 10.2337/diacare.20.11.1717

[10] American Diabetes Association Professional Practice Committee (2024) Management of diabetes in pregnancy: standards of care in diabetes. Diabetes Care 47(suppl 1):S282–S294 38078583 10.2337/dc24-S015 PMC10725801

[11] Feig DS, Donoven LE, Zinman B et al (2020) Metformin in women with type 2 diabetes in pregnancy (MITY): a multicenter, international, randomized, placebo-controlled trial. Lancet Diabetes Endocrinol, Elsevier 8(10):834–844 10.1016/S2213-8587(20)30310-7 32946820

[12] ACOG Practice Bulletins of Clinical Management Guidelines for Obstetrician-Gynecologists (2018) ACOG Practice Bulletins NO. 190: Gestational diabetes mellitus. Obstet Gynecol 131(2):e49–e64 29370047 10.1097/AOG.0000000000002501

[13] Hadlock FP, Harrist RB, Carpenter RJ, Deter RL, Park SK (1984) Sonographic estimation of fetal weight. the value of femur length in addition to head and abdomen measurements. Radiology 150(2):535–540 6691115 10.1148/radiology.150.2.6691115

[14] Society for Maternal-Fetal Medicine (2018) DC.SMFM statement: pharmacological treatment of gestational diabetes. Am J Obstet Gynecol 218:B2–B4 10.1016/j.ajog.2018.01.041 29409848

[15] National Institute for Health and Care Excellence 2015 Diabetes in pregnancy: management of diabetes and its complications from preconception to the postnatal period. NICE guideline (NG3). London: National Institute for Health and Care Excellence (cited 2020 Feb 1).

[16] Balsells M, Garcia-Patterson A, Sola I, Roque M, Gich I, Corcoy R (2015) Glibenclamide, metformin, and insulin for the treatment of gestational diabetes: a systematic review and meta-analysis. BMJ 350:h102 25609400 10.1136/bmj.h102 PMC4301599

[17] Butalia S, Gutierrez L, Lodha A, Aitken E, Zakariasen A, Donovan L (2017) Short- and long-termoutcomes of metformin compared with insulinalone in pregnancy: a systematic review and meta-analysis. Diabet Med 34(27):36 10.1111/dme.13150 27150509

[18] Tarry-Adkins JL, Aiken CE, Ozanne SE (2020) Comparative impact of pharmacological treatment for gestational diabetes on neonatal antrhropometry independent of maternal glycemic control: A systematic review and meta-analysis. PLOS Med 17(5):e1003126 32442232 10.1371/journal.pmed.1003126 PMC7244100

[19] Glueck CJ, Goldenberg N, Wang P, Loftspring M, Sherman A (2002) Metformin during pregnancy reduces insulin, insulin resistance, insulin secretion, weight, testosterone, and development of gestational diabetes: prospective longitudinal assessment of women with polycystic ovary syndrome from preconception through pregnancy. Hum Reprod 17(11):2858–2864 14998944 10.1093/humrep/deh109

[20] Kjos SL, Schaefer-Graf UM (2007) Modified therapy for gestational diabetes using high risk and low risk fetal abdominal circumference growth to select strict versus relaxed maternal glycemic targets. Diabetes Care 30(suppl 2):S200–S205 17596472 10.2337/dc07-s216

[21] Feig DS, Zinman B, Asztalos E, Donovan LE, Shah PS, Sanchez J, Tomlinson G, Murphy KE (2022) Determinants of small for gestational age in women with type 2 diabetes mellitus in pregnancy: who should receive metformin? Diabetes Care 45:1532–1539 35671033 10.2337/dc22-0013

[22] Landon MB, Gabbe SG (2011) Gestational diabetes mellitus. Obstet Gynecol 118(6):1379–1393 22105269 10.1097/AOG.0b013e31823974e2

[23] Brown J, Grzeskowiak L, Williamson K, Downie MR, Crowther CA (2017) Insulin for the treatment of women with gestational diabetes. Cochrane Database Syst Rev 11:CD012037 29103210 10.1002/14651858.CD012037.pub2 PMC6486160

[24] Butalia S, Gutieerre L, Lodha A, Aitken E, Zakariasen A, Donoven L (2017) Short -and long term outcomes of metformin compared with insulin alone in pregnancy: a systematic review and meta-analysis. Diabet Med 34(27):36 10.1111/dme.13150 27150509

[25] Eyal S, Easterling TR, Carr D, Umans JG, Miodovnik M, Hankins GD et al (2010) Pharmacokinetics of metformin during pregnancy. Drug Metab Disposition. 38:833–840 10.1124/dmd.109.031245 PMC2872944 20118196

[26] Nguyen L, Chan SY, Teo AKK (2018) Metformin from mother to unborn child-are there un warranted effects? EBioMedicine 35:394–404 30166273 10.1016/j.ebiom.2018.08.047 PMC6156706

[27] Barbour LA, Feig DS (2019) Metformin for gestational diabetes mellites:progency, perspective, and a personalized approach. Diabetes Care 42:396–399 30787061 10.2337/dci18-0055

